# Tumor Microenvironment-Responsive Magnetotactic Bacteria-Based Multi-Drug Delivery Platform for MRI-Visualized Tumor Photothermal Chemodynamic Therapy

**DOI:** 10.3390/biology13090658

**Published:** 2024-08-25

**Authors:** Feng Feng, Qilong Li, Xuefei Sun, Li Yao, Xiuyu Wang

**Affiliations:** 1 Institute of Process Equipment, College of Energy Engineering, Zhejiang University, Hangzhou 310027, China; fengfeng0896@zju.edu.cn; 2 Institute of Chemistry Chinese Academy of Sciences, Beijing 100190, China; liqilong@iccas.ac.cn (Q.L.); sunxuefei@iccas.ac.cn (X.S.)

**Keywords:** MRI visualization, combination therapy, multi-drug delivery platform, magnetotactic bacteria, enhanced photothermal–chemodynamic therapy

## Abstract

**Simple Summary:**

Cancer cells display elevated reactive oxygen species (ROS) and an altered redox status. For this study, taking advantage of these characteristics, we designed a multifunctional MRI-visualizable multi-drug delivery platform, AMB@PDAP-Fe (APPF), based on magnetotactic bacteria (AMB) for MRI-visualized tumor photo-thermotherapy (PTT) and enhanced chemodynamic therapy (CDT). Upon magnetic navigation, APPF accumulated in the tumor microenvironment. The PDAP-Fe in the composite was reduced by glutathione (GSH) in the tumor microenvironment and produced Fe^2+^, which catalyzed the generation of reactive oxygen species (ROS) through the Fenton reaction and induced tumor cell apoptosis. The magnetosomes in AMB displayed good dual-mode contrast capability in an MRI, which was later used to visualize the process of tumor treatment. Upon NIR light irradiation, the magnetosomes in magnetotactic bacteria immediately converted the light energy into heat energy and the tumor tissue was heated up, thus causing the death of tumor cells. It is noteworthy that the heat generated during the synergistic PTT process (to which PDAP partly contributed) accelerated the releasing of Fe^2+^ from PDAP-Fe, which enhanced the catalytic conversion of endogenous H_2_O_2_ into ·OH, and, thus, achieved synergistic photothermal-enhanced Fenton-reaction-mediated cancer therapy. This study demonstrates the feasibility of developing an MRI-visualizable multi-drug delivery platform from magnetic bacteria, which, compared with other manmade inorganic multi-drug delivery platforms, are lively, self-propelled, and magnetically navigated. This study also highlights the synergistic tumor-curing effect between magnetic bacteria and PDAP-Fe and the importance of understanding the underlying cooperative molecular mechanism between different drugs when scheduling combination therapy.

**Abstract:**

Cancer cells display elevated reactive oxygen species (ROS) and altered redox status. Herein, based on these characteristics, we present a multi-drug delivery platform, AMB@PDAP-Fe (APPF), from the magnetotactic bacterium AMB-1 and realize MRI-visualized tumor-microenvironment-responsive photothermal–chemodynamic therapy. The Fe^3+^ in PDAP-Fe is reduced by the GSH at the tumor site and is released in the form of highly active Fe^2+^, which catalyzes the generation of ROS through the Fenton reaction and inhibits tumor growth. At the same time, the significant absorption of the mineralized magnetosomes in AMB-1 cells in the NIR region enables them to efficiently convert near-infrared light into heat energy for photothermal therapy (PTT), to which PDAP also contributes. The heat generated in the PTT process accelerates the process of Fe^2+^ release, thereby achieving an enhanced Fenton reaction in the tumor microenvironment. In addition, the magnetosomes in AMB-1 are used as an MRI contrast agent, and the curing process is visualized. This tumor microenvironment-responsive MTB-based multi-drug delivery platform displays the potency to combat tumors and demonstrates the utility and practicality of understanding the cooperative molecular mechanism when designing multi-drug combination therapies.

## 1. Introduction

Elucidation of the various defects that underlie cancer has highlighted its complexity and emphasized the inadequacies of single-drug therapies [1,2]. Combining different therapeutics has been proven to be more effective than single-agent therapies. This has shifted cancer treatment from a focus on monotherapy to multi-drug therapy, based on an active cooperation between two or more treatments, which may result in significant super-additive (namely, “1 + 1 > 2”) therapeutic effects [3]. For example, chemotherapeutics, when combined with P-glycoprotein (P-gp) inhibitors or tyrosine kinase inhibitors (TKIs), enhance cytotoxicity [4]. While different drug combinations have continuously proven promising, their curing efficacy remains unsatisfactory, as they cause little to no improvement and often produce severe side effects [5]. This could be attributed to neglecting the differences between each drug when studying their cooperative cytotoxicity. Different drugs have different physicochemical properties, and the differences in their physicochemical properties may lead to dissimilarities in their pharmacokinetics and tissue distribution; their individual toxicity profile might also differ in different tissues [6]. To overcome this problem and to realize the full potential of combination therapy, the research community has focused on developing different strategies to design and engineer drug delivery platforms (DDPs) for the delivery of multiple drugs, as DDPs that are capable of the precise and controlled delivery of drugs mitigate side effects and improve therapeutic efficacy, yielding effective combination therapies [7,8]. Therefore, how to better design multi-drug delivery platforms so that different drugs can be better combined and effectively delivered to the action site at the same time is an important frontier in combined tumor therapy.

Multi-drug delivery platforms that are involved in combined therapy, such as metal oxide nanoparticles [9,10], biomimetic systems [11,12,13] (e.g., cell membrane-coated nanoparticles [13]), and internally or externally responsive nano-systems [14,15,16] (e.g., pH-responsive nanoparticles [15]), generally use passive carriers that lack self-motivation and greatly rely on blood flow to passively reach tumor tissue; because of the complicated extracellular microenvironment of tumor tissue, in fibrosis, for instance, and because of its high internal pressure, accumulation of the therapeutics at the tumor site is less than 1% [17]. Compared with the manmade non-living multi-drug delivery carriers that have been developed in the last few decades, bacteria, as self-propelled, engineerable microorganisms, could be used as an active drug delivery system, considering their auto-mobility, which can help them to migrate into solid tumors and accumulate in both hypoxia and non-hypoxia regions, and considering the presence of multiple reactive molecules or proteins on the membrane, which could allow different concept-based modifications [18].

Magnetotactic bacteria (MTB) are naturally biomineralized bacteria that automatically synthesize magnetosomes. The magnetosomes within MTB are phospholipid bilayer-wrapped magnetite (Fe_3_O_4_) nanoparticle chains or, less commonly, cinerite (Fe_3_S_4_) magnetic iron chains, which greatly enhance the magnetic anisotropy of the nanoparticles and have a magnetic moment of 6 × 10^−15^ Am^2^ [19]. The magnetosomes can quickly respond to external magnetic fields and help the MTB to migrate and accumulate in deep tumor tissues [20,21]. In addition, MTB are often found living in oxic-anoxic regions, and their facultative anaerobic behavior is greatly beneficial for targeting the hypoxia regions in tumor tissues [22]. Combining their living preference for the anoxic region and the magnetite properties of their magnetosome, MTB could be used as an MRI-visualized multi-drug delivery platform. Therefore, compared to other non-living drug-delivery carriers, MTB represent an efficient automotive carrier to deliver multi-drug therapies based on different design concepts. MTB provide several advantages over non-living carriers when used as multi-drug delivery carriers, including improved flexibility of navigation, an improved ability to accumulate in both hypoxia and non-hypoxia tumor regions, and show minimal invasiveness [23]. Furthermore, the magnetosomes in MTB exhibit significant absorption in the near-infrared (NIR) region and can convert light energy into heat energy, which could eventually result in the thermal ablation of cancer. Therefore, MTB can not only be used as an efficient automotive multi-drug nanocarrier in tumor treatment but can also function as an effective tumor killer for use in tumor-related photothermal therapy (PTT).

For this work, we designed a multifunctional multi-drug delivery platform of AMB@PDAP-Fe (APPF) based on magnetotactic bacteria (AMB) for use in MRI-visualized tumor photo-thermotherapy (PTT) and enhanced chemodynamic therapy (CDT). Upon magnetic navigation, APPF accumulated in tumor tissues. The PDAP-Fe in the composite was reduced by glutathione (GSH) in the tumor microenvironment and produced Fe^2+^, which catalyzed the generation of reactive oxygen species (ROS) through the Fenton reaction and, thus, induced tumor cell apoptosis. Upon NIR light irradiation, the magnetosomes in the magnetotactic bacteria immediately converted the light energy into heat energy, and the tumor tissue in the NIR light spot heated up. The PDAP also contributed to the heating-up of the tumor tissue because of its own mild photothermal ability. The combined photothermal effect of AMB-1 and PDAP rapidly elevated the temperature at the tumor site and caused tumor cell death. It is noteworthy that the heat generated during the synergistic PTT process accelerated the release of Fe^2+^ from the PDAP-Fe, which enhanced the catalytic conversion of endogenous H_2_O_2_ into ·OH and, thus, achieved synergistic, photothermally enhanced Fenton reaction-mediated cancer therapy. AMB@PDAP-Fe not only killed the cancer cells by chemotherapy but also greatly increased the sensitivity of tumor cells to laser irradiation for enhanced PTT [24,25], thereby producing bimodal synergistic effects based on chemotherapy-enhanced PTT. This showed that rational therapeutic combinations based on a deep understanding of the underlying molecular mechanisms are potentially powerful in the treatment of cancer.

## 2. Materials and Methods

### 2.1. Materials

Potassium dihydrogen phosphate (KH_2_PO_4_), sodium nitrate (NaNO_3_), succinic acid, L-cysteine, peptone, yeast extract, ethylenediaminetetraacetic acid (EDTA), magnesium sulfate (MgSO_4_·7H_2_O), manganese sulfate (MnSO_4_·4H_2_O), sodium chloride (NaCl), ferrous sulfate (FeSO_4_·7H_2_O), cobalt(II) chloride hexahydrate (CoCl_2_·6H_2_O), calcium chloride (CaCl_2_), zinc sulfate (ZnSO_4_·7H_2_O), copper sulfate pentahydrate (CuSO_4_·5H_2_O), potassium alum (AlK(SO_4_)_2_), boric acid (H_3_BO_3_), sodium tungstate dihydrate (NaWO_4_·2H_2_O), sodium molybdate dihydrate (NaMoO_4_·2H_2_O), nickel chloride hexahydrate (NiCl_2_·6H_2_O), anhydrous sodium selenite (Na_2_SeO_3_), D-tartaric acid, succinic acid, biotin, folic acid, riboflavin, vitamin B12, calcium pantothenate, para-aminobenzoic acid, thioctic acid, nicotinic acid, quinic acid, and agar were purchased from Maclin (Tijuana, Baja California, Mexico). In addition, 2,6-diaminopyridine, ferric chloride hexahydrate, dimethyl sulfoxide (DMSO), hydrogen peroxide (H_2_O_2_), and 3,3′,5,5′-tetramethylbenzidine (TMB) were purchased from Beijing Solarbio, Beijing, China.

### 2.2. AMB-1 Cultivation and Characterization

AMB-1 (5 mL) in their logarithmic growth phase were cultivated in 200 mL sterilized culture medium in a 250 mL culture flask for 72 h and their growth curve was recorded. In 100 μL of deionized water, the resuspended AMB-1 were transferred to a carbon-supported copper membrane and dried. The carbon-supported membrane was subjected to TEM (transmission electron microscope, HT7700, Hitachi, Beijing, China) observation. The 72-h cultured bacterial suspension was placed in a UV-visible spectrophotometer with applied vertical and horizontal magnetic fields. The OD_600_ value of AMB-1 was measured at a wavelength of 600 nm. By changing the direction of the applied magnetic field, the OD_600_ value under the horizontal magnetic field “OD//;” and under the vertical magnetic field “OD⊥” were measured. The ratio of these two values represents the Cmag value.

### 2.3. Preparation of APPF Based on Magnetotactic Bacteria

PDAP-Fe was prepared through Fe (III)-mediated 2,6-2 aminopyridine (DAP) oxidation polymerization. Briefly, 20 mM of FeCl_3_·6H_2_O was dissolved in 100 mL of deionized water. Then, 20 mM of DAP was added to the mixture and stirred at 37 °C for 24 h for polymerization. Subsequently, the mixture was dialyzed and the Fe-PDAP nanoparticle solution was obtained (dialysis membrane for 12 kDa MW). The Fe-PDAP solution was then freeze-dried and stored at 4 °C. Then, 10 mL of the AMB-1 bacterial solution with an OD_600_ value of 0.1 was centrifuged at 3000 rpm for 10 min. The precipitate was resuspended in 5 mL of deionized water and centrifuged again at 3000 rpm for 10 min. This process was repeated three times. The obtained AMB-1 precipitate was dispersed in 0.5 mL of deionized water, after which 100 μL of PDAP-Fe (1 mg/mL) was added before the volume was adjusted to 1 mL. The mixture was incubated at 28 °C and shaken at 100 rpm for 20 min. After centrifugation at 2000 rpm for 15 min, the precipitate was collected and dispersed in deionized water, followed by three rounds of washing at 2000 rpm. The final suspension was resuspended in deionized water and APPF was obtained, which was then stored at 4 °C.

### 2.4. In Vitro Photothermal Performance Testing

The samples were irradiated with an 808 nm laser (1.0 W/cm^2^, 300 s) in a sample cell. The temperature and photographs of the solution were recorded using a near-infrared thermal imaging camera every 30 s. To calculate the photothermal conversion efficiency (η), the TTIS suspension (100 µg/mL, 0.5 mL) was irradiated with the 808 nm laser (1.0 W/cm^2^) until the temperature stabilized. The photothermal performance of different concentrations of APPF was tested. The concentrations were set as follows: 1 × 10^9^ CFU/mL, 1.5 × 10^9^ CFU/mL, 2.0 × 10^9^ CFU/mL, and 4 × 10^9^ CFU/mL, with a volume of 0.5 mL. The effect of the different laser powers (808 nm) on the photothermal performance of APPF was tested. The APPF concentration was set at 1 × 10^9^ CFU/mL, and the laser powers used were 0.5 W/cm^2^, 1.0 W/cm^2^, and 1.5 W/cm^2^. The laser irradiation was performed for 10 min. Finally, the photothermal performance of different groups of materials was tested: PBS, PDAP-Fe, AMB-1, and AMB@PDAP-Fe. The laser power was set at 1.0 W/cm^2^, and laser irradiation was performed for 10 min.

### 2.5. Measurement of GSH-Dependent Iron Release

To measure the release of Fe^2+^ triggered by GSH in APPF, APPF (1 × 10^9^ CFU/mL, 2 mL) was dispersed in phosphate buffer solutions with and without GSH (200 µM). The suspensions were dialyzed against a buffer solution (pH 7.4, 10 mL) for 24 h, with a cutoff value of 12 kDa MW. At specific time intervals, equal aliquots of 1.0 mL dialysate were sampled and replaced with an equal volume of fresh culture medium. The released Fe^2+^ in the buffer solution was collected and mixed with a solution of bathophenanthroline (50 µL, 100 mM), which served as a Fe^2+^ probe (bathophenanthroline reacts with Fe^2+^ to form a complex that exhibits absorbance at 512 nm). The concentration of the released Fe^2+^ was measured using UV-vis absorption spectroscopy.

### 2.6. Intracellular Reactive Oxygen Species (ROS) Detection

The 4T1 tumor cells were seeded in a confocal microscopy culture dish and allowed to adhere overnight. Once the cell density reached confluency, the culture medium was replaced with 2 mL of medium containing the following substances: PBS + GSH (100 µM GSH), H_2_O_2_ (100 × 10^−6^ M), APPF (1 × 10^9^ CFU/mL) + H_2_O_2_ (100 × 10^−6^ M), and APPF (1 × 10^9^ CFU/mL) + GSH (100 µM) + H_2_O_2_ (100 × 10^−6^ M). Different incubation times were set for each group (30 min, 1 h, and 4 h for the APPF + GSH group; 4 h for other groups). The cells were then washed three times with PBS. After incubating the samples with 20 × 10^−6^ M DCFH-DA for 15 min, the cells were observed under a confocal laser-scanning microscope (Olympus-FV1000, Beijing, China).

### 2.7. In Vitro Photothermal Therapy

The 4T1 cells were seeded in a 96-well plate at a density of 2000 cells per well and then incubated overnight. The culture medium was then replaced with 1 mL of medium containing APPF (1 × 10^9^ CFU/mL), and the cells were irradiated with an 808 nm laser (1.0 W/cm^2^) for 10 min. After that, the cells were washed and incubated with fresh medium for an additional 12 h, and cell viability was measured using the CCK-8 kit (Beijing Solarbio).

### 2.8. MRI Measurement

MRI (0.5 T) was used to determine the T_1_ and T_2_ relaxation times of APPF. For T_1_, the following parameters were listed: repetition time (TR) = 6000 ms, number of data = 25, and number of averages (NA) = 2. For T_2,_ the parameters were as follows: TR = 6000 ms, echo time (TE) = 1 ms, echo count = 6000, and NA = 2. The r_1_ and r_2_ relaxivities were calculated using the linear fitting of 1/T_1_ or 1/T_2_ as a function of metal (Fe + Mn) concentration. For the T_1_- and T_2_-weighted MR images, the instrument parameters were set as follows: TR = 100 ms, TE = 18.2 ms, imaging matrix = 192 × 256, slice thickness = 5 mm, field of view (FOV) = 100 mm × 100 mm, and NA = 2. In the in vivo MR imaging experiments, tumor-bearing mice were anesthetized with 100 μL of 10% chloral hydrate through intraperitoneal injection. Then, 200 μL of APPF solution was injected intravenously into the mouse. MR images were acquired before injection and at 1, 5, and 9 h post-injection, respectively.

### 2.9. In Vivo Anti-Tumor Experiment

A volume of 100 µL of 4T1 cell suspension (approximately 4 × 10^5^ cells) was subcutaneously injected into the right hind backs of 6-week-old BALB/c mice (purchased from Beijing Vital River, Beijing, China). Tumor treatment experiments were performed when the tumors reached a volume of approximately 70–100 mm^3^, which was typically 5–7 days following cell injection. When the tumor volume reached approximately 100 mm^3^, the mice were injected with 25 µL of saline solution, AMB-1 (2 × 10^9^ CFU/mL), PDAP-Fe (72.68 µg/mL), or APPF (2 × 10^9^ CFU/mL) around the tumor site. Two hours after injection, the groups that were injected with saline, AMB-1, and APPF were subjected to 808 nm laser irradiation at a power density of 1.0 W/cm^2^ for 10 min. The temperature was recorded using a thermal imaging camera. During the treatment period, the body weight and tumor size of the mice were measured daily. The tumor volume was calculated as V = *π*/6 × L × W^2^, where V is the tumor volume, L is the tumor length, and W is the tumor width. On the 21st day, the mice were anesthetized, and 500 µL of blood was collected from each mouse. The blood was centrifuged at 2000× *g* for 15 min (4 °C) to obtain plasma for biochemical analysis. The mouse tumor tissue was also collected and weighed.

### 2.10. Statistical Analysis

All statistical analysis in this research was conducted using Student’s *t*-test through GraphPad Prism 8. (*), (**), and (***) represent *p* < 0.05, *p* < 0.01, and *p* < 0.001, respectively. *p* < 0.05 was considered significant. All data were expressed as the mean with standard deviation (mean ± SD).

## 3. Results

### 3.1. Preparation and Characterization of APPF

**Figure 1:**
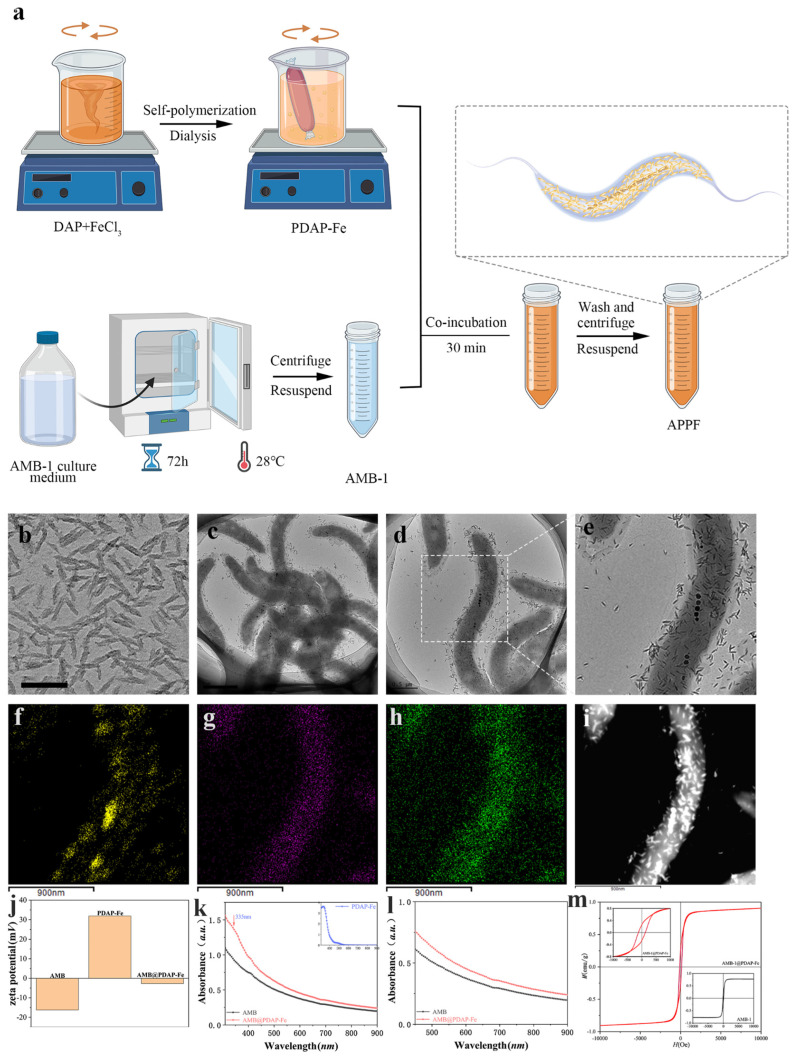
Preparation and characterization of APPF. (**a**) Schematic illustration of APPF preparation; (**b**) TEM image of PDAP-Fe, (**c**–**e**) APPF; (**f**–**i**) elemental mapping of APPF: (**f**) Fe, (**g**) N, (**h**) O and the corresponding dark-field TEM images; (**j**) zeta potentials of AMB-1, PDAP-Fe, and APPF; (**k**) UV-visible absorption spectra of AMB-1, PDAP-Fe, and APPF; (**l**) absorption spectra of AMB-1 and APPF in the infrared region; (**m**) M-H curves of AMB-1 and APPF at room temperature. The scale bar is 50 nm.

The preparation of APPF was conducted as illustrated in Figure 1a. Fe-ion-doped PDAP-Fe was synthesized in aqueous solution, using FeCl_3_ as the oxidant and 2,6-diaminopyridine (DAP) as the precursor [26,27]. Subsequently, purified AMB-1 was co-incubated with PDAP-Fe. The PDAP-Fe rapidly absorbed onto AMB-1 (electrostatic interaction), yielding a multifunctional platform, APPF. Transmission electron microscopy (TEM) images showed that the synthesized PDAP-Fe exhibited shuttle-like structures of approximately 40 nm (Figure 1b). The as-formed APPF retained the morphology of AMB-1, and PDAP-Fe coated the AMB-1 evenly (Figure 1c). The volume of APPF was slightly larger than that of AMB-1 because of the coating. No disruption of the membrane structure of AMB-1 was observed in the magnified images (Figure 1d,e). Because PDAP-Fe was the product of Fe ion-driven DAP polymerization and the use of Fe ions was key to the production of APPF, we analyzed the distribution of Fe elements in APPF using dark-field TEM and surface elemental mapping (Figure 1f–i). The results demonstrated a significant distribution of Fe elements on the surface of APPF, mainly in the PDAP-Fe-formed nanocoating and the magnetosomes of the bacteria. The abundant loading of Fe ions provided a foundation for the efficient catalytic activity of CDT in APPF [28]. To study whether the interaction between PDAP-Fe and AMB-1 was an electrostatic interaction, we analyzed their zeta potentials. AMB-1 displayed a mild negative charge before co-incubation, at approximately −16.9 mV, while PDAP-Fe exhibited a positive charge of around 35.4 mV, due to the presence of amino acid groups in its structure. The zeta potential of APPF dropped to −3.2 mV, indicating that AMB-1 and PDAP-Fe neutralized each other and that the adsorption of PDAP-Fe on AMB-1 was indeed electrostatic absorption (Figure 1j).

To study their light absorption properties, we conducted UV-visible absorption spectroscopy. APPF showed broad absorption in the range of 300–900 nm, with a characteristic absorption peak of PDAP-Fe appearing at 335 nm (Figure 1k), further indicating the effective loading of PDAP-Fe in APPF. Both AMB-1 and APPF exhibited noticeable absorption in the near-infrared wavelength range (Figure 1l), implying the possibility of their being used as photothermal agents. Subsequently, we characterized the magnetic properties of APPF before and after preparation (Figure 1m). The results showed that both AMB-1 and APPF exhibited superparamagnetic behavior, and, due to the presence of magnetosomes, they displayed a certain amount of residual magnetization at low fields, which was consistent with previous reports [29].

### 3.2. APPF Demonstrates Good Photothermal Effects and Induces the Fenton Reaction In Vitro

**Figure 2:**
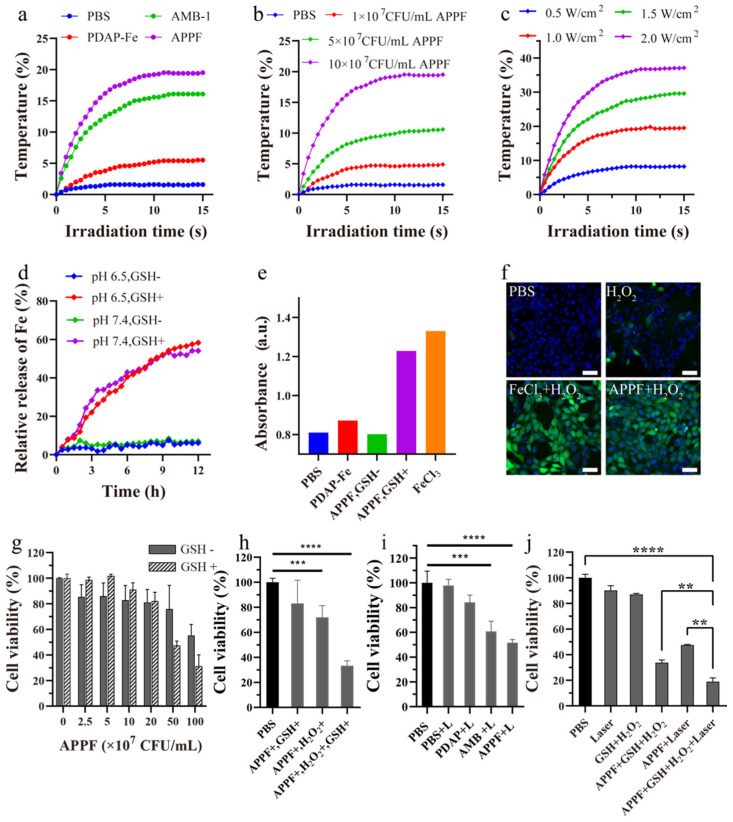
Photothermal performance and Fenton reactions of APPF in vitro. (**a**) Photothermal performance of AMB−1, PDAP−Fe, and APPF under 808 nm laser irradiation; (**b**) the heating curve of APPF with different concentrations under 808 nm laser irradiation; (**c**) the heating curve of APPF under 808 nm laser irradiation at different powers; (**d**) the curve of Fe ions released from APPF under different pH values and with or without GSH; (**e**) UV−vis absorbance of TMB and H_2_O_2_ solutions incubated with APPF for 20 min at room temperature; (**f**) confocal microscope images of DCFH−DA−stained 4T1 cells 3 h after treatment with H_2_O_2_, FeCl_3_ + H_2_O_2_ or APPF + H_2_O_2_ at pH = 6; (**g**) the cytotoxicity of APPF in 4T1 cells with or without GSH; (**h**) the anti−tumor effect of the APPF−catalyzed Fenton reaction; (**i**) the viability of 4T1 cells treated with APPF under 808 nm laser irradiation; (**j**) the in vitro synergistic anti−tumor effect of photothermal therapy and chemodynamic therapy using APPF; 4T1 cells treated with PBS alone were used as a control (100% viability). Unless otherwise specified, the concentration of H_2_O_2_ was 100 × 10^−6^ M and the concentration of GSH was 200 μM. ** *p* < 0.01, *** *p* < 0.001, **** *p* < 0.0001.

The high absorption of APPF in the NIR region enabled us to investigate its photothermal performance, which is typical for PTT applications. As shown in Figure 2a, the PBS group exhibited almost no change in temperature (1.6 °C increase within 10 min of irradiation); the PDAP-Fe group at equimolar concentrations showed a mild photothermal effect (5 °C increase), which failed to reach the therapeutic window for PTT. However, both the AMB-1 and APPF groups demonstrated significant radiation time-dependent temperature elevation, with APPF exhibiting a bigger temperature increase (19.5 °C) than AMB-1 (16.1 °C) at the same concentration (1 × 10^8^ CFU/mL). These results indicated that APPF effectively converted the NIR light energy into heat energy. Apart from the laser irradiation time, concentration and laser power are two other important factors that influence the photothermal performance of APPF and AMB-1 [30]. As shown in Figure 2b,c, APPF exhibited concentration- and laser-power-dependent temperature elevation. At low concentrations, there was almost no significant temperature increase, but, as the concentration increased, the temperature elevation became more pronounced. For example, within 10 min of irradiation, the temperature of APPF at a concentration of 10 × 10^7^ CFU/mL increased by 19.5 °C, while the temperature of the dispersion at a concentration of 1 × 10^7^ CFU/mL increased by only 4.9 °C. Likewise, when the concentration was set (1 × 10^8^ CFU/mL), the temperature of APPF increased along with the increase in the laser power. For example, the temperature of APPF increased by 19.5 °C for the group irradiated with a laser power of 1.0 W/cm^2^, by 37.1 °C for the group irradiated with a laser power of 2.0 W/cm^2^, and by only 8.2 °C for the group irradiated with a laser power of 0.5 W/cm^2^. These results demonstrate that the photothermal behavior of APPF could be precisely controlled by adjusting the irradiation time, laser power, and APPF concentration. Considering that AMB-1 had similar absorption in the near-infrared region and showed nearly identical photothermal characteristics with APPF, it was reasonably expected that AMB-1 had a similar photothermal conversion ability to APPF.

The use of the Fe^2+^/Fe^3+^-catalyzed Fenton reaction to convert H_2_O_2_ into highly reactive ·OH has been widely studied for cancer therapy [31,32]. To investigate the catalytic performance of APPF, we first examined its ability to release Fe^2+^. Due to the higher affinity of Fe^3+^ to S than to N, the PDAP-Fe in APPF was expected to release Fe^2+^ in the presence of GSH [33]. In this study, 200 μM of GSH was added to the APPF suspension to trigger the reduction of Fe^3+^ and the release of Fe^2+^. Because phenanthroline reacts with the Fe^2+^ complex, with products showing a distinct absorption peak at 512 nm [34], we used phenanthroline to test if any Fe^2+^ was released. The results showed that the loaded Fe ions (~58.3%) were released within 12 h in the presence of GSH, while almost no Fe^2+^ release was observed in the absence of GSH (Figure 2d). This indicated that GSH effectively triggered the reduction of Fe^3+^ and the release of Fe^2+^. Importantly, pH had no significant effect on Fe^2+^ release, indicating that APPF specifically responded to GSH to release Fe^2+^. To monitor if the Fenton reaction occurred following Fe^2+^ release, we performed UV-visible light spectroscopy using 3,3,5,5-tetramethylbenzidine (TMB). As shown in Figure 2e, 20 min after APPF addition, the TMB/H_2_O_2_ solution exhibited a measurable increase in absorbance at pH 6.5, similar to the positive control of FeCl_3_, whereas the absorbance of the PBS group remained unchanged. This result demonstrated that APPF selectively responded to GSH, releasing Fe^2+^ with efficient catalytic activity for the Fenton reaction. In tumor cells, a high concentration of H_2_O_2_ exists [35,36], of which Fe^2+^ could catalyze to generate reactive oxygen species (ROS) in order to activate the apoptotic pathway and promote cell death [37]. To test if this process happened with APPF, we used 2,7-dichlorofluorescein diacetate (DCFH-DA), a commonly used ROS probe [38,39], to detect Fe^2+^-catalyzed ·OH generation in 4T1 cells. As shown in Figure 2f, cells treated with PBS or H_2_O_2_ alone exhibited weak fluorescence, while cells treated with APPF and the positive control, FeCl_3_, showed strong green fluorescence. This indicated that APPF effectively catalyzed the generation of ·OH in tumor cells.

The above experiments demonstrated the excellent performance of APPF in performing photothermal and chemodynamic therapy in vitro. Therefore, we further evaluated the anti-tumor effect of APPF at the cellular level. The cytotoxicity of APPF in 4T1 cells was assessed using the Cell Counting Kit-8 (CCK-8) assay. When the concentration of APPF was lower than 2 × 10^8^ CFU/mL, no significant toxicity was observed in the presence or absence of GSH. However, when the concentration of APPF was 5 × 10^8^ CFU/mL, although 4T1 cells with no GSH addition retained a high viability (75.9%), 4T1 cells in the presence of GSH exhibited significantly low viability (47.5%), possibly due to the release of Fe^2+^ that was triggered by GSH (Figure 2g). This result indicated that APPF was biosafe when the concentration was below 2 × 10^8^ CFU/mL. Next, we evaluated the anti-tumor effect of the APPF-mediated Fenton reaction. As shown in Figure 2h, APPF exhibited mild cytotoxicity in the presence of H_2_O_2_ alone. However, APPF exhibited a significant anti-tumor effect in the presence of both GSH and H_2_O_2_; at 1 × 10^8^ CFU/mL, the cell viability decreased by more than 70%. Similarly, under 808 nm laser irradiation, the cell viability of 4T1 cells treated with APPF and AMB-1 decreased significantly, with a reduction of 50% and 40%, respectively (Figure 2h,i), indicating the excellent photothermal (PTT) effect of APPF in vitro. To study if any synergistic anti-tumor effect exists, we investigated the combined anti-tumor effect of PTT and Fenton reaction-mediated chemodynamic therapy (CDT) using APPF at a concentration of 1 × 10^8^ CFU/mL and a laser power of 1 W/cm^2^. As shown in Figure 2j, when the 4T1 cells were treated with APPF, the laser, and H_2_O_2_ in the presence of GSH, over 80% of the cells were killed, indicating a significantly higher anti-tumor effect compared to PTT alone (50%) or the Fenton reaction alone (70%). These results revealed the synergistic anti-tumor effect of PTT and the Fenton reaction when using APPF (Graphpad Prism 8).

### 3.3. APPF Demonstrates Good MRI Capability

**Figure 3:**
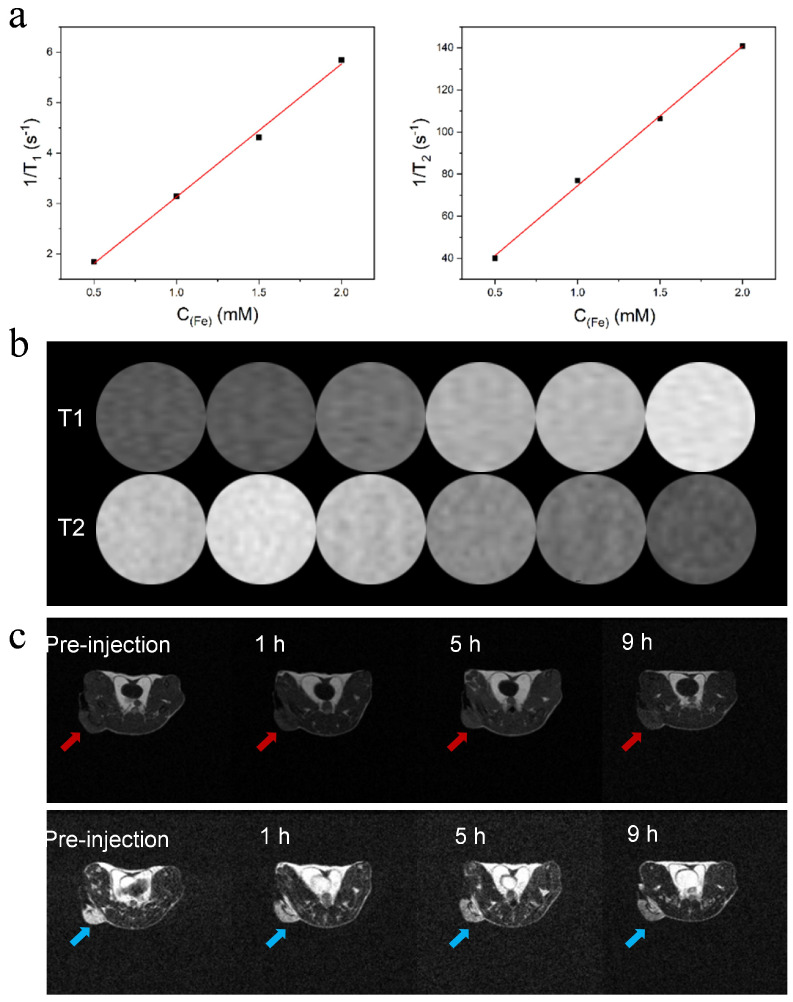
In vitro and in vivo MRI performance of APPF. (**a**) Plots of 1/T_1_ and 1/T_2_ as a function of total metal (Fe) concentrations. (**b**) T_1_−weighted MR images (**top**) and T_2_−weighted MR images (**bottom**) of APPF at different concentrations. (**c**) T_1_− and T_2_−weighted MR images of a 4T1 tumor−bearing mouse before (30 min) and after the intravenous injection of APPF. Tumor regions are highlighted by red and blue arrows.

Magnetic resonance imaging (MRI) is a widely used clinical diagnosis technique offering high spatial resolution and real-time monitoring [40]. MRI contrast agents are commonly employed to improve the contrast between the pathological and normal areas, so as to accurately distinguish the lesion site from normal tissues. To investigate the contrast enhancement of APPF, the longitudinal relaxivity r_1_ and transverse relaxivity r_2_ of APPF were calculated from the linear fitting of the 1/T_1_ and 1/T_2_ plots versus metal concentrations (Figure 3a). The values of r_1_ and r_2_ were estimated to be 4 and 68 mM^−1^ s^−1^, respectively. More importantly, the ratio of r_2_/r_1_ was calculated to be 17, which indicates that APPF can be used as both T_1_ and T_2_ MRI contrast agents. As shown in Figure 3b, APPF presents excellent positive T_1_ and T_2_ contrast enhancement. In T_1_ contrast enhancement, the brightness of the MR images is enhanced with increasing APPF, whereas in T_2_ contrast enhancement, the brightness of the MR images decreases along with the increase in APPF, indicating a clear dose-dependent color change. Next, we investigated the in vivo MR imaging performance of APPF. For this purpose, 4T1 tumor-bearing mice were intravenously injected with 200 μL of APPF solution. Figure 3c shows both T_1_-weighted and T_2_-weighted MR images, taken before and after injection. Clearly, with the increase in time, the T_1_-weighted MR images displayed an increase in brightness, gradually lighting the tumor up (Figure 3c above), whereas T_2_-weighted MR images showed a decrease in brightness (Figure 3c below), confirming that APPF can both increase the T_1_ MRI contrast and decrease the T_2_ MRI contrast in animal models. The signal enhancement was quantified to be 1.2-fold at 9 h post-injection for T_1_ enhancement, and 3.4-fold for T_2_ enhancement at the same hour. The slow increase in signal intensity over time suggested that APPF accumulated in the tumor site. The in vivo-enhanced T_1_- and T_2_-positive signal of APPF makes it a promising MRI contrast agent to facilitate tumor diagnosis and tumor therapy.

### 3.4. Combined Photothermal and Chemodynamic Anti-Tumor Effect of APPF

**Figure 4:**
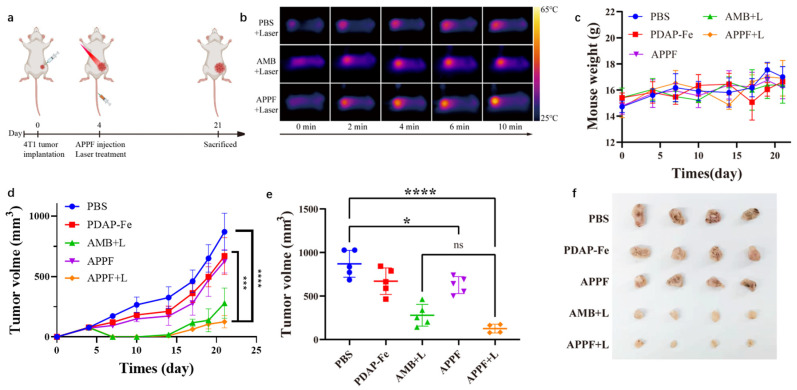
The combined anti-tumor effect of APPF-mediated photothermal therapy and chemodynamic therapy in vivo. (**a**) Schematic illustration of the treatment schedule. (**b**) Thermal images of tumor-bearing mice injected intravenously with PBS, AMB-1, or APPF under 808 nm laser irradiation at the indicated time points. (**c**) Tumor growth curve of mice treated with different protocols. (**d**) The weight change of the mice in different groups throughout the treatment. (**e**) Differential analysis of the tumor volume in different treatment groups. (**f**) Display of the tumor tissue obtained from different treatment groups. The data are shown as mean ± standard deviation (the number of mice in each group was 4 or 5). * *p* < 0.05, *** *p* < 0.001, **** *p* < 0.0001.

**Figure 5:**
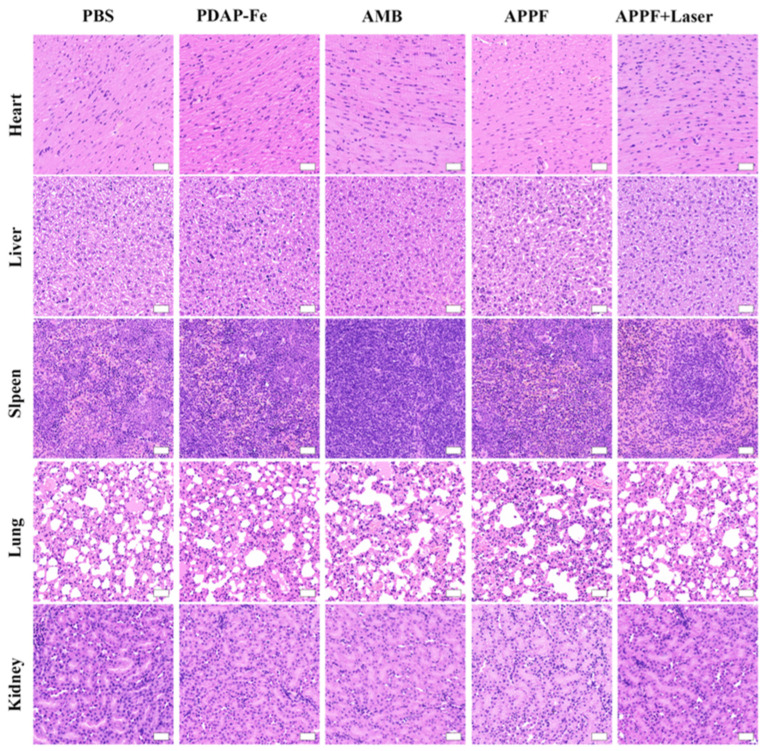
Biosafety assessment of APPF, showing the post-treatment H&E morphology evaluation of the isolated main organs collected from the mice. The scale bar is 100 nm.

After validating the combined anti-tumor effect of APPF at the cellular level and the ability to enhance MR imaging, we evaluated the in vivo anti-tumor effect of APPF. For this purpose, 4T1 tumor-bearing mice were randomly divided into five treatment groups (PBS, PDAP-Fe, APPF, AMB + Laser, and APPF + Laser) and treated according to the scheme shown in Figure 4a. Laser irradiation was performed 2 h after drug injection, and the temperature of the tumor tissue in the mice was recorded. As shown in Figure 4b, compared to the PBS injection group (ΔT = 5.8 °C), mice treated with AMB + Laser exhibited a significant increase in temperature (ΔT = 13.7 °C). Under the same level of laser irradiation, mice injected with APPF showed a greater temperature increase (ΔT = 16.9 °C) at the irradiation site, which indicated that besides AMB, PDAP-Fe also contributed to the thermotherapy effect of APPF. To investigate if APPF had any potential side effects on the tumor-bearing mice, we continuously monitored the body weight of the mice throughout the treatment process. We found that there was no significant change in mouse body weight throughout the entire treatment regimen (Figure 4c), indicating the overall safety of the treatment protocol. More importantly, we continuously monitored the growth of the mice tumors during the treatment process to evaluate the efficacy of different treatment regimens (Figure 4d). In the PBS group, tumor growth was relatively rapid. The PDAP-Fe and APPF groups showed some inhibition of tumor growth, demonstrating the effectiveness of Fenton-reaction-mediated cancer therapy. Strong tumor suppression was observed in mice treated with AMB + Laser, indicating the anti-tumor effect of PTT. As expected, the treatment regimen using APPF + Laser exhibited stronger tumor suppression compared to PTT induced by AMB + Laser or CDT induced by PDAP-Fe or APPF alone, which was attributed to the combined PTT and CDT of APPF treatment. At the end of the treatment, differential analyses of tumor volume were performed among the different treatment groups, and the results were consistent with the tumor growth curve, with the APPF + Laser group showing a significantly smaller tumor volume than the other treatment groups (Figure 4e,f). To verify the presence of any potential adverse reactions, we studied the postmortem histopathology of the major organs (heart, liver, spleen, lungs, and kidneys). Negligible morphological differences were observed in the organs of each group, further supporting the good biocompatibility of the APPF treatment strategy (Figure 5).

## 4. Discussion

Cancer cells display elevated reactive oxygen species (ROS) and altered redox status. Taking advantage of these characteristics, numerous therapeutic techniques have been developed, including chemotherapy and photothermal therapy (PTT). Magnetotactic bacteria (MTB) are naturally biomineralized bacteria that synthesize magnetosomes; they respond to external magnetic fields and help MTB to migrate and accumulate in deep tumor tissues and can be used as an MRI contrast agent [20]. Compared with other photothermal agents (for example, nanoparticles and small molecules), MTB provide several advantages when used as a PTT effector, including MRI visualization, improved flexibility of navigating, an improved ability to accumulate in both hypoxia and non-hypoxia tumor regions, and minimal invasiveness.

Based on the magnetotactic bacterium AMB-1, we developed a multifunctional multi-drug delivery platform, APPF, for MRI-visualized combined photothermal therapy (PTT) and enhanced Fenton-reaction-mediated chemodynamic therapy (CDT). By taking advantage of the high affinity of Fe ions to the nitrogen (N) in PDAP, we prepared PDAP-Fe nanoparticles with a high loading capacity of Fe ions. The doped Fe^3+^ ions were reduced by glutathione (GSH) in the tumor site, releasing highly active Fe^2+^ to catalyze the Fenton reaction, thus initiating the generation of reactive oxygen species (ROS) and the inhibition of tumor growth. Additionally, the magnetite nanoparticles formed inside AMB-1 cells exhibited significant absorption in the near-infrared (NIR) region, enabling the efficient conversion of NIR laser energy into heat energy for PTT-based anti-tumor therapy. The PDAP in APPF also contributed to the PTT behavior of APPF. It is noteworthy that the heat generated during PTT accelerated the release of Fe^2+^ from APPF, which enhanced the Fenton reaction in the H_2_O_2_-rich tumor microenvironment for cancer treatment. This work demonstrates the fabrication of a composite multifunctional platform based on the magnetotactic bacterium AMB-1 through simple electrostatic interactions. It further confirms the synergistic effect of PTT and Fenton-reaction-mediated cancer treatment, providing a new alternative for tumor therapy and a new design strategy for the application of magnetotactic bacteria in cancer treatment.

Moreover, these findings not only influence specific engineering concepts and medical interventional methods but also open up opportunities for the synthesis of new targeted therapeutic, imaging, and diagnostic vectors in future potential clinical applications, while providing further opportunities to enhance the delivery and efficacy of existing nanocarriers. However, the inherent complexity of the AMB-1-based combinational platform requires more effort regarding biocompatibility evaluation, such as the platform’s neurotoxicity, clearance, and pharmacokinetic and biodistribution profiles.

## Data Availability

Data are contained within the article.
